# A cluster randomised controlled trial of two rounds of mass drug administration in Zanzibar, a malaria pre-elimination setting—high coverage and safety, but no significant impact on transmission

**DOI:** 10.1186/s12916-018-1202-8

**Published:** 2018-12-10

**Authors:** Ulrika Morris, Mwinyi I. Msellem, Humphrey Mkali, Atiqul Islam, Berit Aydin-Schmidt, Irina Jovel, Shija Joseph Shija, Mwinyi Khamis, Safia Mohammed Ali, Lamija Hodzic, Ellinor Magnusson, Eugenie Poirot, Adam Bennett, Michael C. Sachs, Joel Tarning, Andreas Mårtensson, Abdullah S. Ali, Anders Björkman

**Affiliations:** 1 Department of Microbiology, Tumor, and Cell Biology, Karolinska Institutet, Stockholm, Sweden; 2 Zanzibar Malaria Elimination Programme, Ministry of Health, Zanzibar, Tanzania; 3 Malaria Elimination Initiative, Global Health Group, University of California San Francisco, San Francisco, USA; 4 Biostatistics Unit, Institute of Environmental Medicine, Karolinska Institutet, Stockholm, Sweden; 5 Mahidol Oxford Tropical Medicine Research Unit, Faculty of Tropical Medicine, Bangkok, Thailand; 6 Nuffield Department of Clinical Medicine, Centre for Tropical Medicine, University of Oxford, Oxford, UK; 7 Department of Women’s and Children’s Health, International Maternal and Child Health, Uppsala University, Uppsala, Sweden

**Keywords:** Mass drug administration, Malaria, Elimination, Low transmission, Dihydroartemisinin-piperaquine, Single low-dose primaquine, Coverage, Adherence, Effectiveness, Safety

## Abstract

**Background:**

Mass drug administration (MDA) has the potential to interrupt malaria transmission and has been suggested as a tool for malaria elimination in low-endemic settings. This study aimed to determine the effectiveness and safety of two rounds of MDA in Zanzibar, a pre-elimination setting.

**Methods:**

A cluster randomised controlled trial was conducted in 16 areas considered as malaria hotspots, with an annual parasite index of > 0.8%. The areas were randomised to eight intervention and eight control clusters. The intervention included two rounds of MDA with dihydroartemisinin-piperaquine and single low-dose primaquine 4 weeks apart in May–June 2016. Primary and secondary outcomes were cumulative confirmed malaria case incidences 6 months post-MDA and parasite prevalences determined by PCR 3 months post-MDA. Additional outcomes included intervention coverage, treatment adherence, occurrence of adverse events, and cumulative incidences 3, 12, and 16 months post-MDA.

**Results:**

Intervention coverage was 91.0% (9959/10944) and 87.7% (9355/10666) in the first and second rounds, respectively; self-reported adherence was 82.0% (881/1136) and 93.7% (985/1196). Adverse events were reported in 11.6% (147/1268) and 3.2% (37/1143) of post-MDA survey respondents after both rounds respectively. No serious adverse event was reported. No difference in cumulative malaria case incidence was observed between the control and intervention arms 6 months post-MDA (4.2 and 3.9 per 1000 population; *p* = 0.94). Neither was there a difference in PCR-determined parasite prevalences 3 months post-MDA (1.4% and 1.7%; OR = 1.0, *p* = 0.94), although having received at least the first MDA was associated with reduced odds of malaria infection (aOR = 0.35; *p* = 0.02). Among confirmed malaria cases at health facilities, 26.0% and 26.3% reported recent travel outside Zanzibar in the intervention and control shehias (aOR ≥ 85; *p* ≤ 0.001).

**Conclusions:**

MDA was implemented with high coverage, adherence, and tolerability. Despite this, no significant impact on transmission was observed. The findings suggest that two rounds of MDA in a single year may not be sufficient for a sustained impact on transmission in a pre-elimination setting, especially when the MDA impact is restricted by imported malaria. Importantly, this study adds to the limited evidence for the use of MDA in low transmission settings in sub-Saharan Africa.

**Trial registration:**

ClinicalTrials.gov, NCT02721186 (registration date: March 29, 2016)

**Electronic supplementary material:**

The online version of this article (10.1186/s12916-018-1202-8) contains supplementary material, which is available to authorized users.

## Background

Global advances in malaria control have led to increased international commitment to malaria elimination [1]. A major challenge in achieving elimination is the identification and targeting of sub-microscopic and asymptomatic malaria infections, which are important for continued malaria transmission in low transmission settings [2, 3]. Mass testing and treatment (MTAT) and mass drug administration (MDA) are two potential strategies for targeting such infections [4]. MTAT involves screening all individuals in a given geographical area and treating those found positive for malaria. MTAT has been evaluated for use in malaria elimination settings, including Zanzibar [4, 5], but has not proven to influence transmission possibly due to low sensitivity of available diagnostic tools such as microscopy and rapid diagnostic tests (RDTs) [4, 6]. MDA is defined as the empiric administration of a therapeutic course of an antimalarial regimen to a defined population at the same time without screening or diagnostic testing prior to administration [7]. MDA has been a historic component of many malaria control and elimination programmes, but was until recently not recommended by the World Health Organization (WHO) due to concerns about efficacy, logistical feasibility, sustainability, and the risk of accelerating drug resistance [4]. However, limitations of currently available diagnostic tools and the development of efficacious antimalarials with transmission-reducing effects, such as artemisinin-based combination therapies and primaquine, have renewed the interest for MDA [7–9]. The WHO now supports MDA as an additional tool in low-endemic regions approaching interruption of transmission [4, 10].

Recent reviews have summarised the findings of MDA studies conducted in different settings in Asia, Africa, and the Americas [7, 8]. These studies employed a wide variety of MDA regimens incorporating different drugs, dosages, timings, and numbers of MDA rounds. In the first review in 2013, only two out of 32 included studies were conducted in areas of low endemicity (≤ 5% prevalence) [7, 11], and only two were designed as cluster randomised controlled trials (CRCTs) [11, 12]. Overall, the quality of evidence from areas of low endemicity was deemed to be very low [7]. In the more recent review, 48 out of 182 included studies had follow-up periods greater than 6 months. Only 12 of these 48 studies, conducted between 1961 and 2004, interrupted transmission for over 6 months post-MDA [8]. Only one of these 12 was conducted in sub-Saharan Africa. The consensus from both reviews is that MDA seems to have an immediate impact on malaria transmission, but only few studies have shown sustained impact beyond 6 months.

More recently, three pilot studies in Southeast Asia have shown over 90% reductions of the *Plasmodium falciparum* reservoir up to 12 months post-MDA [13–15]. In highly endemic villages in Eastern Myanmar, an uncontrolled before-and-after study of monthly MDA with dihydroartemisinin-piperaquine (DP) and single low dose (SLD) of primaquine showed a sustained fivefold decrease in *P*. *falciparum* incidence [16]. A recent CRCT conducted in low transmission areas (< 10% prevalence) in southern Zambia reported a short-term impact 5 months after two rounds of community-wide MDA with DP (odds ratio (OR) 0.13; *p* = 0.04) [17]. Finally, modelling has predicted that high coverage of repeated mass treatment may result in sustained transmission reduction when combined with vector control in low-endemic areas [18–20]. In conclusion, additional empirical evidence through high-quality CRCTs is clearly needed to determine the immediate and long-term impact of MDA, especially in low-endemic settings in sub-Saharan Africa where the goal is malaria elimination.

Zanzibar has, through high vector control coverage and good access to treatment, reached a state of malaria pre-elimination with low and seasonal transmission [21]. However, a persistent reservoir of sub-microscopic and asymptomatic infections remains an important obstacle in achieving elimination [22]. Zanzibar therefore represents an ideal situation to test MDA in the WHO recommended context of malaria elimination. A pilot MDA was conducted in response to a malaria outbreak in Zanzibar in 2013 [23]. Approximately 8800 inhabitants received a single round of MDA with DP. The MDA was well received by the community, with over 90% coverage and self-reported adherence. The impact of the intervention was, however, not monitored. In our present study, a CRCT was conducted to primarily assess the effectiveness and safety of two rounds of MDA with DP given together with SLD primaquine, for reducing seasonal malaria transmission towards its elimination in Zanzibar. Two rounds of MDA were chosen to maintain a balance between cost, feasibility, and impact. Importantly, this study adds to the limited evidence for the use of MDA in low transmission settings in sub-Saharan Africa, a primary goal for MDA [4].

## Methods

### Study design

#### Study setting and population

**Fig. 1:**
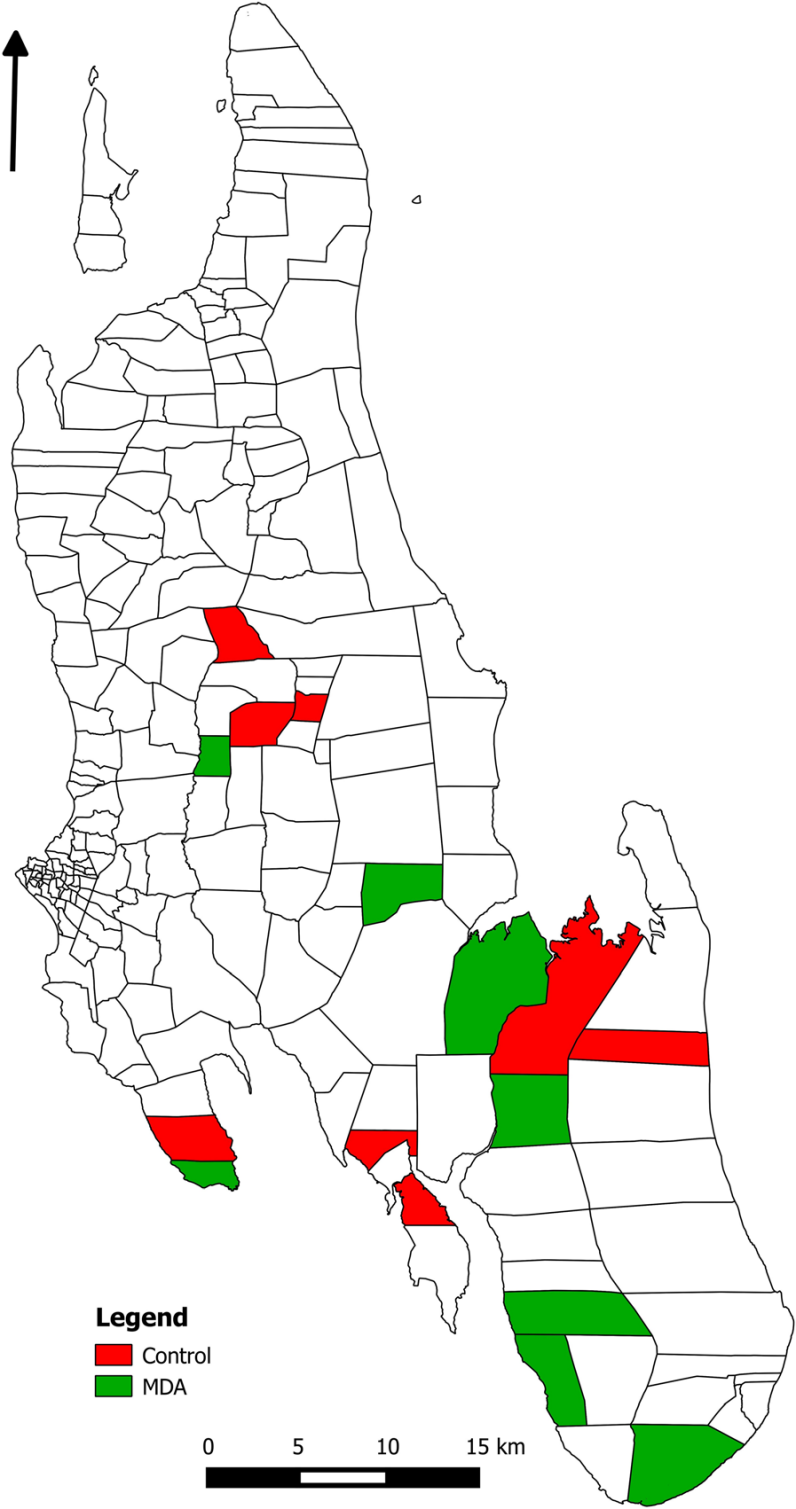
Unguja Island, Zanzibar. The map highlights the 16 shehias included in the study. Shehias randomised to the control arm are shaded in red, and shehias randomised to the intervention arm are shaded in green

A two-armed, open-label CRCT was conducted in 16 shehias (smallest administrative units with typically 2000–5000 inhabitants) in central, south, and west districts on Unguja Island, Zanzibar (Fig. 1). The intervention and control arm each contained eight clusters defined as hotspot shehias with an annual parasite index (API) in 2015 of > 8/1000 population. The API was estimated as the number of confirmed malaria infections reported at health facilities and/or detected during active case detection over the estimated shehia population. The shehia population was based on a consensus survey conducted in 2012 and a population growth of 2.8%. Eligible hotspot shehias were those in the three study districts with a population under 2500.

The entire study population received the standard of care offered by the Zanzibar Ministry of Health and Social Welfare, consisting of diagnosis by RDT or microscopy of clinically suspected malaria in persons presenting to health care centres. Individuals with parasitological diagnosis of malaria at health facilities (hereinafter referred to as “malaria cases”) were treated with first-line drug artesunate-amodiaquine alone. Health care was primarily provided through 15 public health facilities in the 16 shehias.

Universal distribution of long-lasting insecticide-treated nets (LLINs) was conducted in 2012–2013 and 2015–2016 across Zanzibar. The latter distribution was divided in two phases. Approximately one third of the shehias in Unguja received nets in April 2015, including all but two of the study shehias (both in the intervention arm). The remaining shehias received nets in June–July 2016. In addition to these mass campaigns, continuous net distribution has been conducted in all shehias since 2013 targeting pregnant women, children under five, and households with no or worn-out nets.

Zanzibar switched from universal indoor residual spraying (IRS) with pyrethroids to focal targeting of hotspots with carbamate insecticides in 2012. Annual rounds of targeted IRS have since 2014 been conducted with pirimiphos methyl (Actellic® 300CS, Syngenta), a long-lasting insecticide formulation designed for control of pyrethroid-resistant mosquitoes. All study shehias in 2016 and all but 2 shehias (one in each study arm) in 2015 were targeted by IRS.

#### Study intervention

Two rounds of MDA with DP (D-ARTEPP, Guilin Pharmaceutical (Shanghai) Co., Ltd., China) and SLD (0.25 mg/kg) primaquine (Remedica Ltd., Cyprus) were conducted 4 weeks apart in the intervention arm in May–June 2016. Infants under 6 months, women who were either pregnant in the first trimester or whose pregnancy status was unknown (see treatment guidelines, Additional file 1), individuals presenting with severe illness that impaired performance of daily activities, and those having taken antimalarial treatment during the last 14 days were excluded from treatment. In addition, all pregnant women and women breastfeeding infants under 6 months were excluded from treatment with SLD primaquine.

#### Outcome measures

The primary outcome was the cumulative confirmed malaria case incidences in the intervention and control shehias 6 months after the second round of MDA. Confirmed malaria cases were reported in real time through the malaria case notification system (MCN) at health facilities, together with additional information regarding shehia of residence, vector control coverage and uptake, age, sex, and travel history in the last 30 days. Confirmed malaria case incidence was defined as the number of malaria cases in study shehia residents, standardised against the population size estimated at the baseline of the survey to obtain the incidence per 1000 population. The secondary outcome was the community prevalence of malaria infections determined by polymerase chain reaction (PCR) 3 months post-MDA. Additional outcomes included intervention coverage, adherence to the 3-day treatment regimen, occurrence of adverse events, and cumulative incidences 3, 12, and 16 months post-MDA.

### Study procedures

#### Community sensitisation

Community sensitization was conducted prior to study onset to maximise study participation. Village leaders and community members were invited to attend informational meetings held in all 16 shehias; additional meetings were held in the eight intervention shehias before the second round of treatment. Information leaflets were distributed to all households with key messages regarding the study objectives and procedures. Specific information regarding the study drugs and management of possible adverse effects was targeted to the intervention arm only. Local village assistants made public announcements the day before the survey. Automated text messages were sent on two consecutive days after MDA to households where a mobile phone number had been provided to remind participants to take DP doses 2 and 3 and where to go in the case of adverse events.

#### Population enumeration

Population enumeration was conducted in all 16 shehias in association with the first round of MDA (April 30–May 17, 2016). A de facto population approach was used in which all persons sleeping in the household the night before the survey were enumerated (i.e. both permanent and temporary residents). Neighbours were asked to report the number of residents in empty households. Data regarding demographics, uptake of malaria control interventions, known malaria risk factors such as travel history, and eligibility for treatment were collected digitally using Open Data Kit software on tablet computers.

#### Treatment administration and coverage

**Fig. 2:**
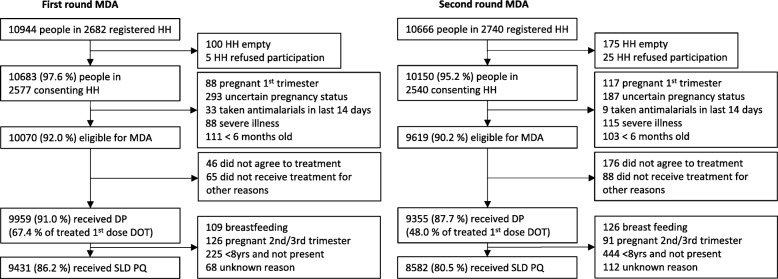
Flow chart of participation in the first and second rounds of MDA. HH household, MDA mass drug administration, DP dihydroartemisinin-piperaquine, DOT directly observed treatment, SLD PQ single low-dose primaquine

Teams of two trained health care workers accompanied by a local guide, distributed treatment to eligible individuals in the intervention shehias during house-to-house visits. Tablet computers were programmed to provide age-based treatment guidelines (see treatment guidelines, Additional file 1) for eligible individuals. Children were given a packet of biscuits for eating after drug intake to prevent abdominal pain, nausea, and vomiting. Persons present during household visits were provided the first drug dose (DP + SLD primaquine) under supervision. The additional two DP doses were left in individual packets with clear instructions for unsupervised intake at home. Labelled packets containing all three doses were left with the head of household for individuals not present. Distribution activities commenced around 8 am and were often completed by 2–4 pm. Children < 8 years who were not present (mainly due to school or after school religious studies) were excluded from receiving the volume-based paediatric dosing of SLD primaquine (Fig. 2). Schoolchildren were therefore asked, with permission from schoolteachers and village leaders, to stay at home on the day of MDA. Efforts were made to revisit households with missing household members later the same day. Coverage was determined as the proportions of the population registered in each round of MDA that received treatment. The proportions of the total population, i.e. the estimated number of people registered in either treatment round (see demographic data collection, Additional file 1), having received zero, one, or two rounds of MDA, were also assessed.

#### Post-MDA surveys and adherence to the 3-day treatment regimen

Post-MDA surveys were conducted in a subset of the population (34 households/shehia) in the intervention arm 7 days after each treatment round. All household members were asked regarding their perception of MDA. Individuals who reported receiving treatment were asked questions regarding adherence and occurrence of adverse events. The self-reported adherence was determined as the proportion of respondents reporting having completed all three doses of DP.

To validate self-reported adherence, finger-prick blood samples to measure day 7 piperaquine blood concentrations were collected from adult participants aged 14 years and older (*N* = 239) during the first post-MDA survey. Blood samples were also collected from adults (*N* = 108) selected from 10 households in each shehia who had taken all three doses of DP under direct observation (observed intake control group). A capillary tube was used to transfer 100 μL blood from the finger to pre-labelled Whatman 31 ETCHR filter papers. Piperaquine whole blood concentrations were measured using solid-phase extraction and liquid chromatography mass-spectrometry at the Department of Clinical Pharmacology, Mahidol Oxford Tropical Medicine Research Unit, Bangkok, Thailand. Quality control standards at 9.00 ng/ml, 40.0 ng/ml, and 800 ng/ml were analysed with each batch of clinical samples to ensure high in-assay precision (i.e. relative standard deviation of < 5%). The reportable range of drug concentrations was 2.4–1150 ng/ml.

#### Active and passive monitoring of adverse events

The occurrence of adverse events was actively monitored using a structured questionnaire during post-MDA surveys. Respondents were asked to report side effects from a list of possible events, together with perceived severity (mild, moderate, or severe) and date of onset and duration. Adverse events were also passively monitored at health facilities by trained health workers for a period of 14 days after each MDA round. Participants were instructed to present to the local health facilities should they experience adverse events such as vomiting, nausea, gastrointestinal upset, rash, fatigue, or dark urine. A standardised form adapted to the study context was used to capture possible serious adverse events following SLD primaquine treatment [24]. In addition, haemoglobin levels and urine colour were measured for assessing the presence of haemoglobinuria or haemolysis (haemoglobin < 5 g/dl or Hillmen colour chart score ≥ 5).

#### Follow-up survey and community prevalence of malaria infection

A follow-up survey, consisting of a tablet-based questionnaire covering uptake of control interventions, risk factors associated with malaria, and perception of MDA, was conducted 3 months post-MDA (Aug 30–Sept 9) in approximately 50% of households in the study area. Finger-prick blood sampling on a Whatman 3MM filter paper for estimating PCR-determined parasite prevalence was conducted in all ages during follow-up (*N* = 9849), as well as at the study baseline (*N* = 7941). Approximately 100 μL of blood was collected on pre-labelled filter papers; molecular analyses were conducted at Karolinska Institutet, Stockholm, Sweden. In brief, DNA was extracted from pools of four samples using the Chelex-100 boiling method with minor modifications (see laboratory protocols, Additional file 1). Extracted pools were screened for *Plasmodium* DNA with two different quantitative PCRs (qPCR): cytochrome b (Cytb) qPCR [25] and 18s-qPCR [26]). Individual samples in pools considered positive by either PCR method were re-extracted and subjected to screening with Cytb-qPCR in baseline samples, or both Cytb-qPCR and 18s-qPCR in follow-up samples. *Plasmodium* species was determined by restriction fragment length polymorphism [25]; 18s-qPCR was repeated in triplicate to estimate parasite densities [22, 26].

### Sample size calculation and randomisation

Sample size calculation for the CRCT was based on the incidence of reported malaria cases during the period May–October 2015. After restricting to the 26 highest incidence shehias with a population under 2500 in the three study districts, the calculated coefficient of variation was 0.35. Using this coefficient of variation, a (harmonic) mean shehia population of 1405, and a baseline incidence of 12/1000, eight clusters in each arm were required to detect an expected 50% reduction in infection incidence with 80% power. Random permutations using a shapefile were conducted to select 16 eligible shehias with as little bordering as possible. Allocation of shehias to each arm was conducted in Stata v.12.1 (StataCorp LP, USA) using computerised block randomisation based on shehia population size and a random seed generator. The estimated population size was approximately 12,000 people per arm.

### Statistical analyses

Analyses were intention-to-treat analyses wherein all individuals in the intervention arm were assumed to have received treatment. Unadjusted comparisons of the cumulative malaria case incidence in the intervention and control shehias were conducted at 3, 6 (primary outcome), 12, and 16 months post-MDA. Analyses were done using Wilcoxon rank-sum test with exact statistics, on cluster summaries of cumulative incidence. Cumulative malaria case incidences before and after MDA were compared by Wilcoxon signed-rank test with exact statistics. Parasite prevalence by PCR was compared between study arms by estimating ORs in logistic regression models using generalised estimating equations (GEE) accounting for clustering by shehia. Additional exploratory analyses included univariate and multivariate analyses of risk factors associated with clinical malaria and asymptomatic infections. Individual malaria case data collected in MCN, on risk factors associated with clinical malaria, were compared with corresponding data collected from the general population in the baseline survey. Risk factors associated with asymptomatic malaria were compared at baseline and follow-up in PCR-positive and PCR-negative persons. Risk factor analyses were conducted by logistic regression using GEE with OR as a measure of association; all variables were included in the model for adjusted odds ratios (aOR). Median day 7 piperaquine concentrations were compared between groups by the Wilcoxon rank-sum test. All analyses were conducted using Stata v.12.0, apart from crude incidence rate ratio that was calculated in OpenEpi [27].

## Results

### Baseline characteristics

In total, 23,251 people living in 5688 households were registered at the study baseline. Household participation rate was high; < 0.5% of household heads refused participation, and 5.4% of households were empty or not occupied at the time of the survey. Baseline characteristics were similar between the two arms (Table 1). There was no significant difference in the pre-intervention annual parasite incidence (*p* = 0.19), but the PCR-determined malaria prevalence was significantly lower in the intervention arm than in the control arm at baseline (OR = 0.31, CI95% 0.15–0.61, *p* = 0.001).

**Table 1:** Baseline characteristics in intervention and control shehias, May 2016. Numbers in brackets represent the range between shehias

	Intervention	Control
Survey characteristics		
Date of survey	30 April–7 May, 2016	9 May–17 May, 2016
Number of households registered	2682 (219–453)	3006 (208–644)
Household participation rate^1^ (%)	96.1 (91.4–99.5)	92.8 (87.9–95.4)
Number people registered (all households)	10,944 (926–1821)	12,307 (935–2542)
Number people in consenting households (*N*; %^2^)	10,683; 97.6%	11,813; 96.0%
Proportion present at time of survey^3^ (%)	77.2 (64.9–84.6)	74.6% (61.8–82.8)
Household characteristics		
House type		
Temporary (% palm leaf structure)	7.0 (2.9–11.3)	4.1 (0.0–11.6)
Semi-permanent (% mud and sticks/stones/coral)	24.3 (12.0–50.7)	26.0 (11.4–40.2)
Permanent (% brick/stone)	68.7 (43.4–84.1)	69.9 (48.2–88.6)
Households in proximity to breeding site^4^ (%)	6.8 (1.7–13.6)	5.8 (0.4–18.4)
Head of households worried about malaria^5^ (%)	54.0 (44.5–60.3)	59.2 (49.8–69.4)
Participant characteristics		
Median age [interquartile range]	19 [8–35]	19 [8–35]
Permanent resident of household (%)	98.1 (96.8–98.7)	98.3 (97.6–99.7)
Percent female (%)	51.5 (49.5–54.2)	51.7 (49.8–55.3)
Main occupation^6^		
Farming (%)	37.7 (10.7–54.5)	42.1 (15.4–60.6)
Fishing (%)	12.3 (0.5–33.2)	12.7 (0.4–30.8)
Other (%)	27.7 (20.2–36.7)	25.5 (11.6–44.0)
Unemployed (%)	24.4 (16.6–30.0)	22.9 (15.0–30.2)
Overnight travel within Zanzibar in last month (%)	3.4 (2.1–5.0)	2.9 (1.1–4.3)
Overnight travel outside Zanzibar in last month (%)	0.5 (0.0–1.0)	0.6 (0.1–1.2)
Overnight travel outside Zanzibar in last 6 months (%)	1.4 (0.4–2.8)	1.5 (0.6–2.3)
Vector control coverage		
Households sprayed in the last 12 months (%)	84.7 (64.3–94.2)	85.1 (77.3–93.8)
Households with at least one mosquito net (%)	85.6 (72.6–97.4)	82.3 (72.0–93.4)
Households with at least one mosquito net per sleeping space (%)	67.0 (45.5–81.6)	62.6 (54.0–75.3)
People with access to a bed net^7^ (%)	74.5 (56.6–90.6)	71.1 (59.1–80.6)
People who slept under a mosquito net last night (%)	74.6 (53.9–92.0)	70.2 (55.8–79.6)
Children under five who slept under a mosquito net last night (%)	79.6 (61.5–96.6)	78.8 (62.7–87.4)
Malaria transmission indicators		
Annual parasite index in 2015 (cases/1000 people)	14.4 (11.1–22.4)	12.7 (8.3–32.8)
Malaria prevalence at baseline (%)	0.8 (0.0–1.6)	2.5 (0.7–4.5)

^1^Households consented/households registered

^2^People in consenting households/people registered

^3^People present/people registered in consenting households

^4^Within 50 m of water assembly

^5^Proportion of head of households that are worried that they or a family member will get malaria

^6^Occupation only recorded for individuals over the age of 18; other occupations are occupations listed in < 5% of the population

^7^This indicator estimates the proportion of the population that could potentially be covered by existing ITNs, assuming that each ITN in a household can be used by two people within that household

### Coverage of MDA

Coverage with DP treatment was 91.0% (range between shehias 87.1–93.4%) and 87.7% (78.3–92.8%) in the first and second rounds, respectively. Coverage with SLD primaquine was 86.2% (82.1–89.2%) and 80.5% (69.8–87.2%) (Fig. 2). Of the total population registered in the intervention shehias (*N* = 12,614), 60.6% (range between shehias 49.5–72.9%) received both rounds of MDA, 18.4% (14.1–24.2%) received round 1 only, 13.6% (7.2–19.5%) received round 2 only, and 7.4% (4.6–10.4%) did not receive any MDA. Hence, 92.6% (89.6–95.4%) of the population were reached with at least one treatment round.

Women with uncertain pregnancy status (10.9% and 7.1% of adult women in each round) were the largest group excluded from treatment with DP (Fig. 2). Children under 8 years of age who were not present (9.1% and 19.4% of children < 8 years in each round) were the largest group excluded from treatment with SLD primaquine. Among eligible individuals in each round, only 0.5% and 1.8% refused treatment. The most common reason for refusing treatment was fear of side effects (reported by 50.4% of refusals). In the second round, more participants requested to take the medicine in the evening to avoid experiencing side effects. This resulted in fewer participants (48.0% vs. 67.4% in the first round) having taken the first dose under observation.

### Adherence to the 3-day treatment regimen and adequate drug exposure

**Fig. 3:**
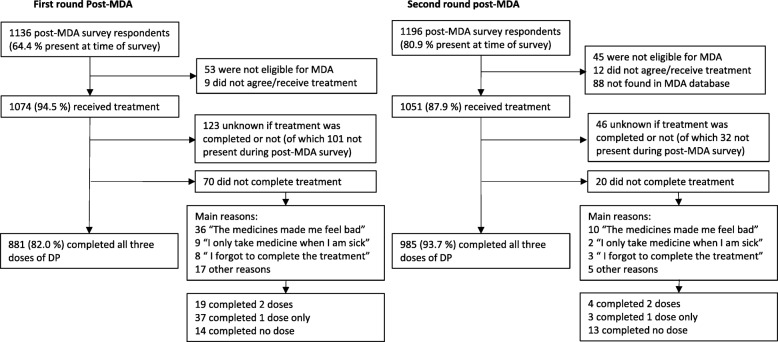
Flow chart of self-reported adherence after the first and second rounds of MDA

Self-reported adherence to the 3-day treatment regimen was 82.0% (range between shehias 71.9–88.6%) and 93.7% (83.7–99.3%) for rounds 1 and 2, respectively (Fig. 3). The main reason for not completing the treatment was experiencing side effects (50.1% of non-completed treatments). The self-reported adherence corresponded with day 7 piperaquine concentrations at the group level (Table 2). There was no significant difference in the median concentrations between the observed intake control group and those reporting full adherence (*p* = 0.19), whilst median drug concentrations were significantly lower in those reporting non-adherence (*p* < 0.001). However, among adults who claimed full adherence, 7.4% (16/215) showed piperaquine concentrations below the limit of quantification indicating incomplete treatment intake (see Additional file 2: Figure S1). Taking into account this overestimation of adherence, an estimated 69.1% and 76.1% (mean 72.6%) of the registered population were covered by adequate drug exposure (i.e. having received and completed the full treatment course) in rounds 1 and 2, respectively.

**Table 2:** Day 7 piperaquine concentrations by adherence status

	Median drug concentration ng/ml [IQR]	*p* value^1^
Observed intake control group (*N* = 94)^2^	128.5 [53.5–189.0]	Reference
Non-observed treatment intake (*N* = 236)^3^	95.5 [34.9–186.0]	0.051
Self-reported adherence		
Full adherence (*N* = 215)	107.0 [41.0–192.0]	0.187
Non-adherence (*N* = 21)	30.4 [2.9–69.6]	*< 0.001*
Self-reported non-adherence		
Completed two doses (*N* = 8)	57.8 [3.3–100.7]	*0.034*
Completed one dose (*N* = 11)	36.8 [2.9–60.9]	*0.001*
Completed no doses (*N* = 2)	7.1 [<LLOQ–12.9]	*0.017*

^1^Calculated by the two-sample Wilcoxon rank-sum (Mann-Whitney) test against the observed intake control group. Significant *p* values are set in italics

^2^Ten samples with drug concentration falling below the limit of quantification (<LLOQ), three samples with reports of vomiting within 30 min after drug intake, and one sample with self-reported non-adherence were excluded from the control group

^3^Three samples excluded due to improper sample conditions

### Safety and tolerability of MDA

Among post-MDA survey respondents who received treatment, 147/1268 (11.6%; range between shehias 5.7–23.8%) and 37/1143 (3.2%; 0.6–8.8%) reported at least one adverse event after the first and second rounds, respectively. In addition, there were 85 and 29 reports of adverse events passively identified at health facilities after rounds 1 and 2. Nausea and vomiting (33.1% of all reports), stomach pain and diarrhoea (18.9%), and dizziness, headache, and fatigue (23.5%) were the most commonly reported adverse events (see Additional file 3: Table S1). Across all adverse events, 44.1% were perceived by survey respondents as mild, 52.0% as moderate, and 0.5% as severe. No MDA-associated deaths or other clinically serious adverse event was reported.

In post-MDA and the follow-up surveys, 1761/1786 (98.6%) and 8115/8966 (90.5%) of present respondents expressed willingness to participate in future MDAs. Even among those who reported adverse events, 146/151 (96.7%) would participate in MDA again.

### Impact of MDA on malaria transmission

#### Confirmed malaria case incidence

**Fig. 4:**
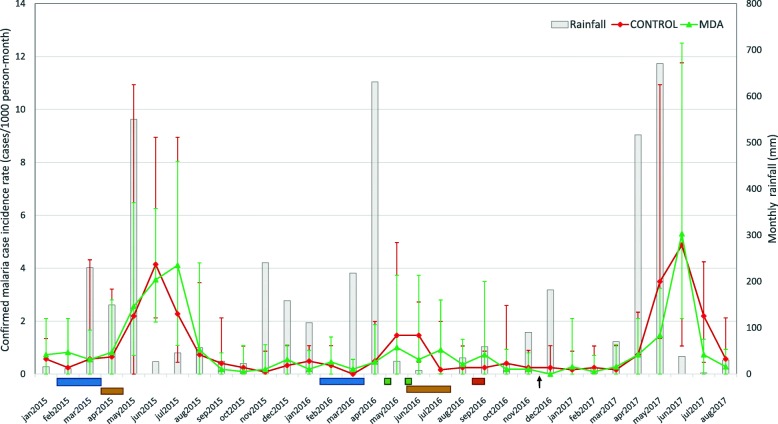
Confirmed malaria case incidence rates as reported in MCN before and after MDA. Error bars represent the range in monthly incidence rates in the control (red) and intervention (green) shehias. Horizontal bars represent the monthly rainfall on Unguja according to the Tanzanian Meteorological Agency Zanzibar Office. The blue bars under the *x*-axes represent the timing of IRS with Actellic®300CS, which is conducted annually in Feb–March in hotspot shehias. The yellow bars represent the two phases of the universal LLIN distribution in April 2015 and June–July 2016. The green bars indicate the timing of the two rounds of MDA (30 April–7 May and 28 May–4 June, respectively). The orange bar indicates the timing of the follow-up survey (30 Aug–9 Sept), and the primary endpoint of the study (30 Nov) is marked out with a black arrowhead

No difference in cumulative malaria case incidence was observed between the control and intervention arms 6 months after MDA (4.2 and 3.9 per 1000 population, respectively; corresponding to a crude incidence rate ratio of 0.94 CI95% 0.63–1.44). Neither was there a difference in cumulative malaria incidence at 3, 12, and 16 months after MDA, or when only locally acquired infections were considered, i.e. excluding those reporting overnight travel to mainland Tanzania in the last month (Table 3). However, there was a 62.6% reduction in cumulative malaria incidence from 10.9 to 4.1 per 1000 population (*p* < 0.001) across both study arms in 2016 (Fig. 4). This reduction was observed across all of Unguja Island in 2016 (see Additional file 2: Figure S2). There was no apparent difference in annual rainfall profiles 2015–2017, although the seasonal rains in April–June 2016 were of shorter duration.

**Table 3:** Cumulative malaria case incidence at 3, 6, 12, and 16 months after MDA

	Malaria cases		Cumulative incidence (cases/1000 population; range between shehias)		
Time period	Intervention (*N* = 10,944)	Control (*N* = 12,307)	Intervention	Control	*p* value
Baseline					
6 months (May–Nov 2015)	128	126	11.7; 7.7–16.1	10.2; 6.4–32.8	0.130
12 months (May 2015–April 2016)	150	146	13.7; 9.8–19.6	11.9; 7.5–36.8	0.105
Post-MDA					
3 months (May–Aug 2016)	31	41	2.8; 0.0–10.3	3.3; 0.9–8.0	0.721
6 months (May–Nov 2016)^1^	43	52	3.9; 0.0–13.1	4.2; 2.1–8.0	0.442
12 months (May 2016–April 2017)	58	71	5.3; 0.0–13.1	5.8; 3.1–10.4	0.382
16 months (May 2016–Aug 2017)	143	208	13.1; 5.4–28.0	16.9; 6.7–31.8	0.130
Post-MDA cases without travel history					
3 months (May–Aug 2016)	18	23	1.6; 0.0–9.3	1.9; 0.0–8.0	0.716
6 months (May–Nov 2016)	22	29	2.0; 0.0–11.2	2.4; 0.9–8.0	0.277
12 months (May 2016–April 2017)	31	41	2.8; 0.0–11.2	3.3; 1.1–8.0	0.315
16 months (May 2016–Aug 2017)	109	160	10.0; 5.4–26.1	13.0; 2.7–30.8	0.279

^1^Primary outcome

#### PCR-determined prevalence of *Plasmodium* infection

No difference in PCR-determined parasite prevalence was observed between the intervention and control shehias in the follow-up survey (OR = 1.0, CI95% 0.5–2.0, *p* = 0.94) (Table 4). Similar findings were observed after excluding individuals with reported overnight travel (OR = 1.0, CI95% 0.5–2.1, *p* = 0.94). *P*. *falciparum* was the predominant species, followed by *P*. *malariae* and *P*. *ovale*, with mean parasite densities around 10 parasites/μL (Table 4). Some 3303 individuals were screened by PCR in both baseline and follow-up surveys. Four individuals were positive for malaria by PCR in both surveys; all four were residents of control shehias. Among the 82 PCR-positive individuals in the follow-up survey in the intervention shehias, 45 (54.9%) had received both treatment rounds, 17 (20.7%) had received either round 1 or 2, and 20 (24.4%) had received no treatment.

**Table 4:** PCR-determined prevalence of *Plasmodium* infection at baseline and during follow-up surveys

	Baseline			Follow-up		
	Intervention	Control	Total	Intervention	Control	Total
Prevalence *n*/*N* (%; range between shehias)	31/4042 (0.8; 0.0–1.6)	95/3875 (2.5; 0.7–4.5)	126/7917 (1.6; 0.0–4.5)	82/4896 (1.7; 0.5–3.9)	71/4905 (1.4; 0.5–4.0)	153/9801 (1.6, 0.5–4.0)
Prevalence without travel history *n*/*N* (%; range between shehias)	28/4042 (0.7; 0.0–1.6)	90/3875 (2.3; 0.7–4.5)	118/7917 (1.5; 0.0–5.0)	79/4896 (1.6; 0.6–3.9)	68/4905 (1.4; 0.5–4.1)	147/9801 (1.5;0.5–4.0)
Malaria species						
*P. falciparum* (*N*; %)	22; 71.0%	64; 67.4%	86; 68.3%	61; 74.4%	47; 66.2%	108; 70.6%
*P*. *malariae* (*N*; %)	2; 6.5%	17; 17.9%	19; 15.1%	7; 8.5%	11; 15.5%	18; 11.8%
*P*. *ovale* (*N*; %)	4; 12.9%	1; 1.1%	5; 4.0%	5; 6.1%	7; 9.9%	12; 7.8%
*P*. *vivax* (*N*; %)	1; 3.2%	0; 0.0%	1; 0.8%	1; 1.2%	0; 0.0%	1; 0.7
Mixed infections (*N*; %)^1^	2; 6.5%	12; 12.6%	14; 11.1%	6; 7.3%	6; 8.5%	12; 7.8%
Undetermined (*N*; %)	0; 0.0%	1; 1.1%	1; 0.8%	2; 2.4%	0; 0.0%	2; 1.3%
Parasite densities						
Geometric mean p/μL [IQR]^2^	8 [2–21]	8 [< 1–50]	8 [< 1–43]	11 [< 1–57]	3 [< 1–21]	6 [< 1–35]
Samples <LLOQ (*N*; %)^3^	6; 19.4%	29; 30.5%	35; 27.8%	43; 52.4%	30; 42.2%	73; 47.7%

^1^Mixed infections were either *P*. *falciparum* + *P. malaria* (88.5% of mixed infection) or *P*. *falciparum* + *P*. *ovale* (11.5% of mixed infections)

^2^Parasite densities below the limit of quantification (1 p/μL) were set as 0.5 p/μL; IQR = interquartile range

^3^<LLOQ = below lower limit of quantification, i.e. samples that were positive in cytb-qPCR but negative in the 18s-qPCR for quantification

#### Risk factors associated with clinical and asymptomatic malaria infection

Among clinical cases, being 15–24 years old, male, and having travelled outside of Zanzibar were all strongly associated with increased odds of infection in both the intervention and control shehias (Table 5). Among confirmed malaria cases in the intervention and control shehias, 26.0% and 26.3% reported recent travel outside Zanzibar, respectively (aOR ≥ 85; *p* ≤ 0.001). In addition, residing in a household covered by IRS and use of a mosquito net the night before the survey were associated with lower odds of infection in the intervention shehias but not in the control shehias.

**Table 5:** Univariate and multivariable analysis of risk factors associated with clinical malaria infections

	Intervention shehias					Control shehias				
	Survey population (*n*; %)^1^	Malaria cases (*n*; %)^2^	OR (CI95%)	aOR^3^(CI95%)	*p* value	Survey population (*n*; %)^1^	Malaria cases (*n*; %)2	OR (CI95%)	aOR3 (CI95%)	*p* value
Residing in households sprayed in the last 12 months										
No	1316; 12.5	18; 23.4	1 (Ref)	1 (Ref)	–	1437; 12.3	24; 18.1	1 (Ref)	1 (Ref)	–
Yes	9218; 87.5	59; 76.6	0.4; 0.3–0.7	0.5; 0.2–0.9	*0.018*	10,205; 87.7	109; 82.0	0.6; 0.4–0.9	0.6; 0.4–1.0	0.074
Residing in household with at least one mosquito net										
No	1283; 12.2	9; 11.7	1 (Ref)	1 (Ref)	–	1739; 14.9	16; 12.0	1 (Ref)	1 (Ref)	–
Yes	9251; 87.8	68; 88.3	1.0; 0.5–2.0	2.8; 0.9–9.1	0.085	9903; 85.1	117; 88.0	1.3; 0.8–2.2	2.6; 1.2–5.5	*0.015*
Residing in households with at least one mosquito net and/or sprayed in the past 12 months										
No	293; 2.8	6; 7.8	1 (Ref)	1 (Ref)	–	200; 1.7	3; 2.3	1 (Ref)	1 (Ref)	–
Yes	10,241; 97.2	71; 92.2	0.3; 0.1–0.7	0.4; 0.1–1.9	0.262	11,442; 98.3	130; 97.7	0.7; 0.2–2.0	0.5; 0.1–2.1	0.356
Age										
< 5 years	1622; 15.4	4; 5.2	1 (Ref)	1 (Ref)	–	1785; 15.3	10; 7.5	1 (Ref)	1 (Ref)	–
5–14 years	2725; 25.9	22; 28.6	3.3; 1.1–9.4	3.7; 1.2–11.1	*0.019*	3104; 26.7	30; 22.6	1.7; 0.8–3.6	2.1; 1.0–4.4	0.064
15–24 years	1895; 18.0	27; 35.1	5.7; 2.0–16.4	4.4; 1.5–13.1	*0.009*	2034; 17.5	42; 31.6	3.8; 1.8–7.7	3.7; 1.7–8.0	*0.001*
> 25 years	4292; 40.7	24; 31.2	2.2; 0.8–6.4	1.4; 0.5–4.3	0.510	4719; 40.5	51; 38.4	1.9; 1.0–3.8	1.6; 0.8–3.2	0.228
Sex										
Female	5440; 51.6	26; 33.8	1 (Ref)	1 (Ref)	–	6028; 51.8	50; 37.6	1 (Ref)	1 (Ref)	–
Male	5094; 48.4	51; 66.2	2.1; 1.3–3.4	2.0; 1.2–3.2	*0.009*	5614; 48.2	83; 62.4	1.8; 1.3–2.6	1.7; 1.1–2.5	*0.009*
Travel in last month										
No travel	10,152; 96.4	54; 70.1	1 (Ref)	1 (Ref)	–	11,262; 96.7	95; 71.4	1 (Ref)	1 (Ref)	–
Within Zanzibar	336; 3.2	3; 3.9	1.7; 0.5–5.3	2.3; 0.7–7.6	0.160	325; 2.8	3; 2.3	1.1; 0.3–3.4	1.1; 0.3–3.7	0.838
Outside of Zanzibar	43; 0.4	20; 26.0	87.5; 46.4–165.1	103.4; 49.8–214.9	*< 0.001*	55; 0.5	35; 26.3	75.8; 42.3–136.0	85.0; 4.2–159.8	*< 0.001*
Reported use of a mosquito net the previous night										
No	2606; 24.7	29; 37.7	1 (Ref)	1 (Ref)	–	3430; 29.5	45; 33.8	1 (Ref)	1 (Ref)	–
Yes	7928; 75.3	48; 62.3	0.5; 0.3–0.8	0.5; 0.3–1.0	*0.033*	8212; 70.5	88; 66.2	0.8; 0.6–1.2	0.7; 0.5–1.1	0.167

^1^Healthy individuals at study baseline: *N*_(Intervention shehias)_ = 10,534; *N*_(Control shehias)_ = 11,642; 194 (0.9%) individuals excluded due to missing data, and 126 (0.6%) excluded for being positive for malaria by PCR

^2^Malaria cases diagnosed at health facilities during the study period May 2016–August 2017: *N*_(Intervention shehias)_ = 77; *N*_(Control shehias)_ = 133; 141 (40.2%) individuals excluded due to missing data

^3^All variables were included in the model of adjusted OR; significant *p* values are presented in italics

Similar associations were observed among asymptomatic infections. At study baseline (see Additional file 3: Table S2a), being 15–24 years old, male, and having travelled outside of Zanzibar in the last 6 months was associated with an increased odds of PCR-detected infection. Residing in households covered by IRS was associated with lower odds of asymptomatic infection. These associations were, however, not as prominent in the follow-up survey (see Additional file 3: Table S2b). In the intervention arm, having received the first (aOR = 0.35; CI95% 0.14–0.86, *p* = 0.02) or both rounds of MDA (aOR = 0.52; CI95% 0.29–0.93, *p* = 0.03) were the only factors significantly associated with reduced odds of infection. The association with only having received the second round of treatment was not significant (aOR = 0.80; CI95% 0.37–1.73, *p* = 0.57). In the control shehias, being 15–24 years old and residing in a household covered by vector control (either IRS or LLIN) were associated with increased and decreased odds of infection 3 months post-MDA, respectively.

## Discussion

Two rounds of MDA were implemented in a population of over 10,000 people in areas considered hotspots in Zanzibar. High intervention coverage and adherence (> 80%) was achieved in each treatment round, and MDA was well tolerated and accepted by the community. Despite successful implementation, no difference in malaria transmission was observed between the intervention and control arms in this pre-elimination setting.

Multiple rounds of MDA with high intervention coverage (i.e. over 80%) are deemed necessary when MDA is used to reduce transmission or eliminate malaria [8, 10]. Coverage is generally determined based on the amount of drugs dispensed and the number of persons targeted in each treatment round. This method may, however, overestimate treatment coverage if missing persons and mobile populations are not correctly accounted for [4]. In our study, population sizes were estimated from a census survey conducted in 2012 and IRS survey data from 2016. Similar numbers were obtained at the study baseline enumeration (data not shown), suggesting that the majority of the study population had been recorded. The coverage in each treatment round was 91.0% and 87.7%. High coverage is, however, only effective if an adequate number of people correctly complete the full course of antimalarial treatment [10]. Adherence is especially an issue when treatment is provided to individuals who are not sick. Adherence measurements mostly rely on self-reporting, but this may be subject to recall bias or over reporting. We therefore validated the self-reported adherence using day 7 piperaquine blood concentrations. This enabled an overall mean estimate regarding effective coverage (i.e. the proportion of the population completing the full treatment course) of 72.6% in each round.

The high coverage and compliance achieved in this study may partly be due to the familiarity of MDA as an intervention through its previous use in control and elimination of schistosomiasis and lymphatic filariasis [28, 29], as well as historic and more recent use in malaria control [23, 30]. Community engagement to build awareness of MDA for asymptomatic malaria and partnerships between researchers, local volunteers, and authorities are also factors noted to contribute to high intervention uptake [23, 30–34]. These factors were considered in the community sensitisation that was conducted prior to the study start. In addition, over half of the head of households still recognise malaria as a health concern (Table 1) despite substantial reductions in malaria morbidity and mortality in Zanzibar, potentially adding to the high uptake of the intervention. Another important component for achieving adequate adherence is the safety and tolerability of the treatment regimen [10]. Pharmacovigilance in this study was planned to ensure training, detection, reporting, management, and follow-up of adverse events by both passive and active surveillance. In line with other studies [13, 14, 16, 34–37], MDA with DP and SLD primaquine was deemed safe, with some transient adverse events and no reports of clinically serious adverse events. In addition, acceptability of the intervention was high with over 90% of survey respondents expressing willingness to participate in future MDAs.

Although high coverage and compliance were achieved, no significant impact on transmission was observed, although having received at least the first MDA was partly protective against asymptomatic infection 3 months post-MDA (aOR = 0.35; *p* = 0.02). Previous studies evaluating the impact of MDA have had varying results [7, 8, 13, 15–17, 30]. Overall, MDA has mostly shown short-term impact on malaria transmission, and only a few studies have provided sustained results [14–16, 38]. Recent studies have shown MDA to have an added effect in areas already experiencing a decline in the malaria burden, when deployed together with enhanced early diagnosis and treatment and supporting interventions that target malaria vectors [13–17]. The study in Zambia is the only previous CRCT showing impact of MDA in a low-endemic area in sub-Saharan Africa [17]. This study reported a reduction in malaria prevalence 5 months after two rounds of MDA with DP, albeit with weak statistical significance (aOR 0.13, CI95% 0.02–0.92, *p* = 0.04).

The optimal transmission scenarios and drug intervention regimens for producing a sustained impact with MDA thus remain largely unknown, and it remains unclear when MDA may be of most benefit in the context of malaria elimination [39]. DP has been suggested as a suitable option for MDA, in view of its good efficacy, long post-treatment prophylaxis, and good tolerability [10]. The addition of SLD primaquine is recommended to further reduce the transmissibility of *P*. *falciparum* gametocytes in areas of low transmission [10, 40]. The number of treatment rounds required to obtain a sustained effect of MDA is however unclear. A single year of two rounds of MDA with an effective coverage of 70% is estimated to provide 14–35% reduction in *P*. *falciparum* prevalence 2 years after MDA [18]. Modelling suggests that increased number of rounds improves the effectiveness, with a greater sustained impact of MDA if continued over 2 years rather than one. The aim of successive rounds is total coverage, i.e. reaching people who were initially missed and people who were treated in the previous rounds but may have been re-infected after MDA [10, 18]. Adding a third treatment round with 70% effective coverage in models only improved effectiveness if additional people were reached who had not previously received treatment [18]. We estimated a mean effective coverage of 72.6% in each treatment round, with 60.6% of the population having received both rounds and 92.6% of the population having received at least one round. Whether or not this coverage is sufficient remains unclear. Perhaps, higher effective coverage of at least two consecutive rounds of MDA is required to provide a long enough prophylactic period to protect against reinfection from infected mosquitoes (i.e. covering a full man-mosquito-man cycle) in a population.

High coverage of consecutive MDA rounds may be especially important in low-endemic areas where imported malaria cases (which may not be affected by MDA) are expected to have a greater relative contribution to transmission [18]. In Zanzibar, the proportion of clinical malaria cases reporting travel has increased in recent years, clearly indicating imported malaria to be an important driver of remaining transmission (Björkman et al. submitted). In the present study, over a quarter of clinical malaria cases reported overnight stay outside of Zanzibar in the last month (Table 5) compared to less than 1% in the general population (Table 1). We therefore, suggest that two rounds of MDA in a single year may not be sufficient to have a sustained impact on transmission in a pre-elimination setting, especially when the impact of MDA is restricted by imported malaria.

Another possible explanation for the lack of impact on transmission could be the timing of the MDA. Modelling has predicted less influence on malaria transmission if MDA is conducted during peak transmission [18, 19]*.* It is therefore recommended in areas of seasonal transmission that MDA be deployed immediately before the start of the main transmission season [4, 9, 10, 14, 18]. The onset of this study was delayed due to political elections, and due to difficulties in importation and registration of the study drugs. The first round of MDA was conducted right at the beginning of the high transmission season and the second round during peak transmission (see Additional file 2: Figure S3). Having received the first round of treatment was indeed associated with reduced odds of PCR-detected malaria infection in the follow-up survey, whilst having only received the second round of treatment was not (see Additional file 3: Table S2b). These data suggest that there may have been a short and transient effect of MDA on local transmission, which had already been diluted when the follow-up survey was conducted 3 months post-MDA. In addition, the difference in the timing of the baseline surveys in the intervention and control shehias (see Additional file 2: Figure S3) may partly explain the difference in malaria prevalence at the study baseline (Table 1). This difference may however also be due to chance since the potential for imbalance across treatment groups despite randomisation is greater in a relatively small number of clusters.

Importantly, the ability to assess the true effectiveness of MDA in this study was affected by the overall decline in malaria transmission that occurred across Unguja island in 2016 (Fig. 4 and Additional file 2: Figure S2). The study was powered to detect a 50% drop in cumulative malaria incidence from 12/1000 to 6/1000 population in the intervention arm during the 6 months following MDA. However, the observed cumulative incidence (4.2/1000) in the control arm was lower than predicted (Table 3). Assessing the impact of interventions in low transmission settings is problematic, not only because it is difficult to achieve great enough power, but also because transmission may be geographically and temporally heterogeneous from year to year. Several other studies have also reported a decline in transmission across study arms [11, 13, 15, 17]. These studies, including the CRCT conducted in Zambia, have largely attributed these declines to the continuing effects of additional interventions such as high coverage with vector control and strong case management and surveillance. It has been argued that these additional interventions are a prerequisite for implementing MDA [17], but when all interventions are introduced simultaneously, the resulting large reductions in incidence may potentially mask the additional impact of MDA. In Zanzibar on the other hand, high vector control coverage, strong malaria case management, and malaria surveillance have been in place since 2008. Actellic®300CS has been in use since 2014 in the annual targeting of malaria hotspots with IRS, and continued high uptake of bed net usage has been reported in this study as well as elsewhere (Björkman et al., submitted). These additional interventions can therefore not explain the large decline in transmission observed across Zanzibar in 2016. Another more likely explanation for the reduction in transmission is the year-to-year fluctuations in climate [41]. Despite there not being any obvious difference in the total rainfall, the rains in 2016 were short and intense and stopped early with a very dry May compared to 2015 and 2017 (Additional file 2: Figure S2). Overall, these general declines in transmission across study arms highlight the importance of a cluster randomised study design when evaluating the impact of MDA on malaria transmission [17, 30, 39].

## Conclusions

MDA was implemented with high coverage, adherence, and tolerability in Zanzibar. Despite this, no significant impact on malaria transmission was observed. The findings suggest that two rounds of MDA in a single year may not be sufficient for a sustained impact on transmission in a pre-elimination setting, especially when the impact of MDA is restricted by imported malaria. Importantly, this study adds to the limited evidence for the use of MDA in low transmission settings in sub-Saharan Africa.

## Additional files

Additional file 1: Supplementary methods. Treatment guidelines, demographic data collection, and laboratory protocols. (DOCX 766 kb)

Additional file 2: **Figure S1.** Capillary whole blood piperaquine concentrations at day 7 post-dose, stratified by self-reported adherence status. **Figure S2.** Number of clinical malaria cases as reported through the malaria case notification system from January 2015 to Sept 2017 in Unguja. **Figure S3.** Weekly number of clinical malaria cases as reported in the Malaria Early Epidemic Detections System in Unguja 2016. (PDF 414 kb)

Additional file 3: **Table S1.** Adverse events reported during post-MDA surveys (active detection) and at health facilities (passive detection) after mass treatment with DP and SLD primaquine. **Table S2a.** Risk factors associated with asymptomatic malaria infection at the baseline of the study. **Table S2b.** Risk factors associated with asymptomatic malaria infection during the follow-up survey three months after completed MDA. (XLSX 30 kb)

## Abbreviations

<LLOQ: Below lower limit of quantification
aOR: Adjusted odds ratios
API: Annual parasite index
CI95%: 95% confidence intervals
CRCTs: Cluster randomised controlled trials
Cytb: Cytochrome b
DOT: Directly observed treatment
DP: Dihydroartemisinin-piperaquine
GEE: Generalised estimating equations
HH: Household
IQR: Interquartile range
IRS: Indoor residual spraying
LLINs: Long-lasting insecticide-treated nets
LLOQ: Lower limit of quantification
MCN: Malaria case notification system
MDA: Mass drug administration
MTAT: Mass testing and treatment
OR: Odds ratio
*P*. *falciparum*: *Plasmodium falciparum*
PCR: Polymerase chain reaction
PQ: Primaquine
qPCR: Quantitative PCR
RDTs: Rapid diagnostic tests
SLD: Single low dose
WHO: World Health Organization
