# Supplementary material for: A cluster randomised controlled trial of two rounds of mass drug administration in Zanzibar, a malaria pre-elimination setting—high coverage and safety, but no significant impact on transmission
Source: BMC Med. 2018 Dec 10;16:215. doi: 10.1186/s12916-018-1202-8 (PMC6287359; doi:10.1186/s12916-018-1202-8)
Supplement: Supplementary file 1 — Supplementary methods. Treatment guidelines, demographic data collection, and laboratory protocols. (DOCX 766 kb) [file 12916_2018_1202_MOESM1_ESM.docx]

**Supplementary methods**

**1. Treatment guidelines**

**
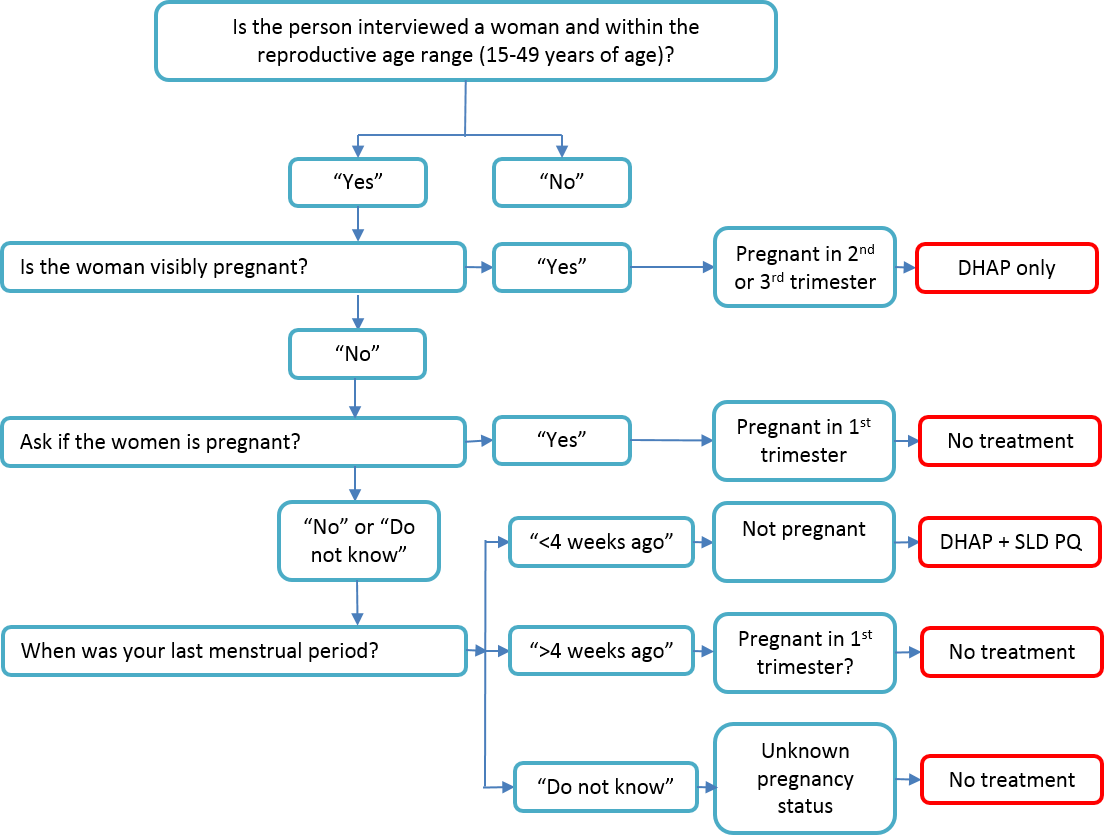
**

**Figure 1: Algorithm used for determining pregnancy status.**

**Table 1: Age based dosing and administration of DHAp and SLD Primaquine.**

| **Age [weight bracket]** | **D-ARTEPP**  **(40mg/320mg)** | **Primaquine**  **(7.5mg)** |
| --- | --- | --- |
| **6 months -1 year [5-9.9kg]** | ½ tablet for 3 days | 2 cc solution*, 2 mg [0.40-0.22 mg/kg] |
| **2-7 years [10-20kg]** | 1 tablet for 3 days | 4 cc solution*, 4 mg [0.40-0.20 mg/kg] |
| **8-13 years [21-40kg]** | 2 tablet for 3 days | One tablet, 7.5 mg [0.36-0.19 mg/kg] |
| **14 years and above [>40kg]** | 3 tablet for 3 days | Two tablets, 15 mg [<0.38 mg/kg] |

* Paediatric dosing of SLD primaquine was done by dissolving 7.5mg tablets in water and then dosing by volume [1] Primaquine tablets were crushed using a mortar and pestle and suspended in drinking water to obtain a 1mg/ml solution. Prepared solutions were kept in glass containers in the refrigerator for up to 3 days. Healthcare workers took aliquots of the solution in clean plastic containers to the field on a daily basis. The age based volume of the solution was measured using a clean syringe per child, and administered directly to the child’s month.

**2. Demographic data collection**

At the study baseline, each household was provided a uniquely labelled and barcoded household participant sheet, on which each household member was listed, together with age and a unique, barcoded personal identification number. Open Data Kit software was used to enter the information into tablet computers, together with data regarding demographics, uptake of malaria control interventions, known malaria risk factors such as travel history, and eligibility for treatment. The participant sheet was photographed on the tablet and left in the household for upcoming surveys. Data was uploaded daily to a local server. Data cleaning was conducted in several stages using STATA v.12.0 (StataCorp LP, USA); all editing was logged in .do files.

1. Data was cleaned on a daily basis for simple errors that could be corrected. Open ended questions that were answered in Swahili (e.g. additional reasons for refusing treatment) were translated to English. Duplicate IDs were corrected by checking electronic copies of consent forms. At the end of each survey cleaned databases from each day were appended to each other and additional cross checking of each variable was conducted.
2. Databases from different surveys were then merged in stages using the household ID (HHID) for the household database and the Person ID (PID) for the database containing individual data. The HH database was merged on a daily basis during the second round of MDA. Over 100 households did not merge on HHID in some shehias. Reasons for houses not matching included: 1) the household was only registered in one of the two surveys 2) the household was empty or refused participation in one of the surveys and did therefore not have a participant sheet 3) the household had miss-placed the participant sheet and was therefore given a new HHID and PIDs. HH that did not merge on HHID were matched based on 1) telephone number of head of household, 2) name of head of household, 3) GPS location. None of these variables were reliable for matching since the telephone number and head of household can vary depending on who was present at the time of the household visit, and the GPS was not accurate enough to match households in clusters. In total 388/2682 (14.5 %) HH registered in the first round and 446/2740 (16.3 %) of HH registered in the second round could not be matched. HH in the follow-up survey were only included if they had a known HHID; 30/3107 (1.0 %) of HH could still not be matched.
3. After the second round of MDA was complete, the entire first and second round databases containing individual data were merged on PID. PIDs that did not merge were then matched by 1) Name and new HHID (i.e. people in households that had been given a new HHID in the second round, and that had been manually matched during the merging of the HH database, could then be matched by name and age) 2) same/similar name and similar age on a shehia level (i.e. manual searches in database sorted by name and age for each shehia). Manual matching by age and name was time consuming and not reliable seeing both variables can vary depending on who was asked at the time of the survey. Approximately 25% of the mismatches in PID were resolved. In total 2079/10683 (19.5 %) of people registered in the first round and 1546/10150 (15.2 %) of people registered in the second round, and 1309/13397 (9.8 %) of people in the follow up survey could not be matched based on PID, shehia, name and age.

**NOTE:** In order to have the most conservative estimate of total population coverage, all unmatched PIDs were assumed to belong to unique individuals, even though this was not likely to be the case.

**3. Laboratory protocols: screening of samples for *Plasmodium* DNA**

3.1.1 Chelex-100 DNA extraction of pooled samples (adapted from Hsiang 2010 [2])

**Preparation of pooled samples**

Materials:

- PerkinElmer 1296-071 DBS Puncher (DELFIA® Dried Blood Spot Puncher) with a Ø 3.2 mm head
- Dried blood spots (DBS) prepared on Whatman 3MM filter paper
- Clean Whatman 3MM filter paper
- Sterile and free DNA/RNA-DNase/RNase 96 well tissue culture plates with lid
- Gloves, permanent marker
- 1.5 mL microfuge tubes (safe cap)

Pool four samples by punching two Ø 3.2 mm punches from each of four samples into the same well of a tissue culture plate (total 8 punches per well) using the DBS Puncher. In between each sample, clean the head of the puncher with two Ø 3.2 mm punches of clean Whatman 3MM filter paper collected in a second “waste” plate. Include two *P. falciparum* positive controls at 10 p/µL and 1 p/µL (2 positive punches + 6 negative punches) and one negative control (8 punches of *Plasmodium* negative blood) in each 96-well plate.

**Chelex-100 DNA extraction of pooled samples**

Materials:

- Pooled punches of dried blood spots
- Sterile 0.2 % high grade Saponin (Sigma-Aldrich) in 1X Phosphate Buffered Saline (PBS): Prepare 1X PBS + 0.2 % saponin in required quantity (e.g. Add 2 g Saponin to 1 L of 1X PBS). Sterilize by filtration. Stored up to one month at 4-8°C.
- 10 % Chelex® 100: 200-400 mesh, sodium form (Bio-rad). Prepare 10 % Chelex-100 in Molecular grade water (Sigma-Aldrich) in 50 ml tube (e.g. add 5 g Chelex into 50 ml water). Mix thoroughly, transfer aliquots into 1.5 mL microfuge tubes whilst Chelex is suspended in solution. Store at 4-8°C.
- 1.5 mL safe cap microfuge tubes, 0.6 mL microfuge tubes, fine tip tweezers, dry heat block at 95°C, shaker, centrifuge, racks, vortex, pipettes, filtered pipette tips (1 mL, 200 µL and 10 0µL), sterile 96 well plates or 0.2 ml tubes for storage of extracted DNA, sealing foil for plates, permanent marker, gloves, 4°C refrigerator, -20°C freezer. Optional: repeater pipette

1. Transfer with a fine tip tweezer all eight punches from each pool into a 1.5mL safe cap microfuge tube. Clean tweezers between samples by rinsing in two consecutive beakers containing distilled water, followed by a beaker of 70% ethanol and finally 99% ethanol. Dry tweezers on clean paper.
2. Using the regular or repeater pipette, dispense 1 mL of 0.2 % saponin in PBS into each microfuge tube. Vortex each tube separately, assuring that punches move freely. Incubate 5 minutes on shaker at room temperature.
3. Remove PBS + Saponin with a pipette (1mL tips). Centrifuge tubes for 5 seconds and remove remaining PBS + saponin from the tubes with a 200 µL pipette.
4. Add 1mL of PBS to each tube (no saponin), invert tubes to mix, again assuring that punches move freely. Incubate 30 minutes on shaker at room temperature.
5. As above, remove PBS with 1ml pipette. Centrifuge tubes for 5 seconds, and remove remaining PBS from the tubes with a 200µL pipette.
6. Transfer 120 µL of 10% Chelex to each sample. Vortex or invert the Chelex every three or four transfers since Chelex settles quickly.
7. Extract the parasite DNA by incubating tubes for 10 minutes in a 95°C heat-block. Vortex the samples before, and gently flick the tubes after incubation.
8. After incubation, centrifuge tubes for 3 minutes at 12,000 rpm.
9. Transfer as much as possible (at least 40µL) DNA elute into 96 well plates. Store the plates short term (1-2 nights) at 4-8°C, otherwise freeze at -20°C until use.

3.1.2 Chelex 100 DNA extraction for single samples

**Preparation of single samples**

Materials: As above

Punch two Ø 3.2 mm punches from each sample into the same well of a tissue culture plate using the DBS Puncher, as described above. Include two *P. falciparum* positive controls at 10p/µL and 1p/µL (2 positive punches from each) and one negative control (2 punches of *Plasmodium* negative blood) in each 96-well plate.

**Chelex-100 DNA extraction of single samples**

Materials: As above

1. Transfer with a fine tip tweezer the 2 punches from each sample into a 1.5mL safe cap microfuge tube as above.
2. Using the regular or repeater pipette, dispense 800µL of 0.2% saponin in PBS into the microfuge tubes. Vortex each tube separately, assuring that punches move freely. Incubate 5 minutes on shaker at room temperature.
3. Remove PBS + Saponin with a pipette (1 mL tips). Centrifuge tubes for 5 seconds and remove remaining PBS + saponin from the tubes with a 200 µL pipette.
4. Add 800 µL of PBS to each tube (no saponin), invert tubes to mix, assuring that punches move freely in the microfuge tube. Incubate 30 minutes on shaker at room temperature.
5. As above, remove PBS with 1 ml pipette. Centrifuge tubes for 5 seconds, and remove remaining PBS from the tubes with a 200 µL pipette.
6. Transfer 100 µL of 10 % Chelex to each sample. Vortex or invert the Chelex every three or four transfers since Chelex settles quickly.
7. Extract the parasite DNA by incubating tubes for 10 minutes in a 95°C heat-block. Vortex samples before, and gently flick the samples after incubation.
8. After incubation, centrifuge tubes for 3 minutes at 12000 rpm. Meanwhile, label one sets of 0.6 mL microfuge tubes for transfer, and one set of 0.2 mL tubes for storage of the extracted DNA.
9. Transfer as much solution as possible from the spun tubes to the first set of 0.6 ml microfuge tubes with a 200 uL filtered-tip, not worrying if a small amount of Chelex is carried over.
10. Spin tubes for 5 minutes at 12000 rpm and then transfer the final, white-to-yellowish supernatant (avoiding the Chelex pellet) to the final set of 0.2 ml tubes. Store extracted DNA short term (1-2 nights) at 4-8°C, otherwise freeze at -20°C until use.

3.2.1 Screening for Plasmodium DNA and estimating parasite densities with 18s-qPCR (adapted from Kamau 2011 [3])

Materials:

- Chelex-100 extracted DNA
- CFX96 Touch^TM^ Real Time PCR Detection System (Bio-Rad)
- Ssso Advance probe based Supermix (Bio-Rad), stored at -20°C
- Forward primer (5’-GCT CTT TCT TGA TTT CTT GGA TG-3’), Reverse primer (5’-AGC AGG TTA AGA TCT CGT TCG-3’) and probe (5’:6-FAM-ATG GCC GTT TTT AGT TCG TG-3’) published in Kamau 2011 [3]. **Note!** The probe is very light sensitive and sensitive to freezing and thawing and should therefore be aliquoted in amber microtubes.
- Nuclease free H_2_O, nuclease free 96 well plates, nuclease free microtubes (2 mL), amber microtubes (0.6mL), single and multichannel pipettes (different volumes), filtered pipette tips, clear sealing foil for plates, gloves, permanent marker.

1. Do 18s-qPCR immediately after Chelex-100 extraction, or the following day (in which case store extracted DNA at 4-8°C overnight).
2. Screen each pool/sample with a single reaction of 18s-qPCR in 96-well plates.
3. Prepare master mix according to Table 2. Master mix should be prepared for 106 reactions if using repeater pipette. Homogenize the master mix gently by turning the tube upside down.
4. After adding the master mix and DNA to the 96-well plate, close the plate with a transparent seal for qPCR. Centrifuge the plate to remove any bubbles. Run PCR programme in Table 2 monitoring florescence signal at end of each 60°C step.
5. Cq values below 40 with smooth amplification curve are considered positive.

**NOTE:** Screening of single samples with 18S-qPCR was not done for samples collected at baseline of the study due to a contamination issue that arose during pooling. This issue was resolved before commencing with the samples collected in the follow-up survey.

**3.2.2 Parasite density quantification**

18S-qPCR was repeated in triplicate in single samples considered positive in 18s and/or Cytb-qPCR. An inter-plate calibrator, consisting of 10^-5^ ng/µl of plasmid containing the 18s-qPCR amplicon, and a negative control were included in each 96 well plate. Parasite densities were estimated against a standard curves generated by Chelex-extracted, 10-fold serial dilutions of laboratory cultured 3D7 *P. falciparum* spotted on filter paper. The standard curves were run in triplicate on a separate plate together with the same inter-plate calibrator. The mean parasite densities generated from two independent standard curves were used as a final estimate of parasite density.

| **Table 2: Master mix for 18s-qPCR** | | | | | |
| --- | --- | --- | --- | --- | --- |
|  | **96-well plates (20µL voume)** | | **PCR-program** | | |
|  | **1 sample (µL)** | **106 samples (µL)** |  |  |  |
| **Nuclease free H_2_O** | 5 | 530 | 95°C | 3 min |  |
| **KamauF (100 pmol/µl)** | 0.05 | 5.3 | 95°C | 15 sec | x45 |
| **KamauR (100 pmol/µl)** | 0.05 | 5.3 | 60°C | 60 sec |  |
| **KamauP (100 pmol/µl)** | 0.025 | 2.65 |  |  |  |
| **Bio-Rad master mix** | 10 | 1060 |  |  |  |
| **Master mix** | 15 | 1603 |  | | |
| **Template DNA** | 5 |  |  |  |  |
| **Final volume** | 20 |  |  |  |  |

3.3.1 Screening for Plasmodium DNA and determining parasite species with Cytb-qPCR (Xu et al 2015, [3])

**Pooled samples**

Materials:

- Chelex-100 extracted DNA from pooled samples, stored at -20°C.
- CFX384 Touch^TM^ Real Time PCR Detection System (Bio-Rad)
- SsoAdvanced^TM^ Universal SYBR® Green Supermix (Bio-Rad), stored in -20°C
- PlasMtR forward primer (5’-TGG TAG CAC AAA TCC TTT AGG G-3’) and PlasMtR reverse primer (5’-TGG TAA TTG ACA TCC AAT CC-3’) published by Xu 2015 [4].
- Nuclease free H_2_O, Nuclease free 384 well plates, nuclease free tubes (15 mL), single and multichannel pipettes, filtered pipette tips, clear sealing foil for plates, gloves, permanent marker.

1. Screen each pool with a single reaction of cytb-qPCR [4] in 384-well plates.
2. Prepare master mix according to Table 3. For 384 well plates the mastermix should be prepared for 480 reactions to be able to use the electronic multichannel pipette. **Note:** The final reaction volume is 15µL in 384 well plates, with 3µL of template DNA.
3. After adding the master mix and DNA to the 384-well plate, carefully close the plate with a transparent seal for qPCR. Centrifuge the plate to remove any bubbles. Run PCR programme in Table 3. Positive samples are determined by gel electrophoresis (see below).

**Single samples**

Materials:

- Chelex-100 extracted DNA from single samples
- Positive controls consisting of DNA extracted from *P. falciparum*, *P. vivax*, *P. malariae*, *P. ovale*
- CFX96 Touch^TM^ Real Time PCR Detection System (Bio-Rad)
- SsoAdvanced^TM^ Universal SYBR® Green Supermix (Bio-Rad), stored in -20°C
- PlasMtR forward primer (5’-TGG TAG CAC AAA TCC TTT AGG G-3’) and PlasMtR reverse primer (5’-TGG TAA TTG ACA TCC AAT CC-3’) published by Xu 2015 [4].
- Nuclease free H_2_O, nuclease free 96 well plates, nuclease free tubes (2 mL) single, multichannel pipette, filtered pipette tips, clear sealing foil for plates, gloves, permanent marker.

1. Do Cytb-qPCR immediately after Chelex-100 extraction of single samples, or the following day (in which case store extracted DNA at 4-8°C overnight).
2. Screen each sample with a single reaction of cytb-qPCR [4] in 96-well plates, with 20µL of PCR reaction and 5µL of template DNA.
3. Prepare master mix according to Table 3. After adding the master mix and DNA to the 96-well plate, carefully close the plate with a transparent seal for qPCR. Centrifuge the plate to remove any bubbles. Run PCR programme in Table 3. Positive samples are determined by gel electrophoresis. Species identification in PCR positive samples is done by RFLP on PCR amplified products [4] (see below).

| **Table 3: Master mix for Cytb-qPCR** | | | | | | | |
| --- | --- | --- | --- | --- | --- | --- | --- |
|  | **384-well plates (15µL volume)** | | **96-well plates (20µL volume)** | | **PCR-program** | | |
|  | **1 sample (µL)** | **480 samples (µL)** | **1 sample** | **106 samples (µL)** |  |  |  |
| **Nuclease free H_2_O** | 4.42 | 2121,6 | 5 | 530 | 95°C | 4 min |  |
| **MtF (100 pmol/µl)** | 0.04 | 19,2 | 0.05 | 5,3 | 95°C | 15 sec | x45 |
| **MtR (100 pmol/µl)** | 0.04 | 19,2 | 0.05 | 5,3 | 60°C | 90 sec |  |
| **Bio-Rad master mix** | 7.5 | 3600 | 10 | 1060 |  |  |  |
| **Master Mix** | 12 | 5760 | 15 | 1600.6 | **Amplicon: 432 bp** | | |
| **Template DNA** | 3 |  | 5 |  |  |  |  |
| **Final Volume** | 15 |  | 20 |  |  |  |  |

**3.3.2 Electrophoresis of Cytb-qPCR products**

Materials:

- Cytb-qPCR amplified products
- Horizontal Electrophoresis system
- 5X Green GoTaq® buffer (Promega) or 6X Loading Dye (Fermentas)
- UltraPure™ Agarose
- 1X and 0.5X TBE
- GelRed™ Nucleic Acid Gel Stain (Bio-Rad)
- GeneRuler^TM^ 100bp DNA Ladder (Thermo-Scientific)
- Molecular Imager® Gel Doc^TM^ XR+ with Image Lab^TM^ Software (Bio-Rad)
- Flasks (500 or 250 mL), single and multichannel pipettes (different volumes), non-filtered tips, sealing foil for plates, gloves, permanent marker, weighing scale, microwave

1. When the qPCR is complete mix PCR products with 5µL of 5X Green GoTaq® buffer (Promega) or 6X Loading dye (Fermentas) prior to gel electrophoresis. 5µL of PCR product from each pool should be resolved in 1.5% agarose (Ultra pure^TM^) with 1X GelRed staining.
2. Heat the Agarose in 1X TBE in the microwave until dissolved. Once the mixture is at ~60°C add GelRed and pour into the gel casting tray with combs.
3. In each row of the gel, add 1-5µL of GeneRuler^TM^ 100bp DNA Ladder, depending on stock concentration in 1 or 2 wells (at the beginning and in the middle of each row).
4. Run the gels until the bands are separated sufficiently for correct identification by size.
5. Photograph gel in Bio-Rad Molecular Imager® Gel Doc^TM^ XR+ using the Image Lab^TM^ Software

**3.3.3 Protocol *Plasmodium* species identification by RFLP**

Materials:

- Positive Cytb-qPCR amplified PCR products (samples and controls)
- FspBI enzyme (Thermo Scientific)
- HpyCH4V enzyme (New England Biolabs)
- (AluI enzyme (New England Biolabs))
- (Csp6I enzyme (Thermo Scientific))
- Nuclease free H_2_O, nuclease free 96 well plates, nuclease free microtubes (1.5 or 2 mL), single and multichannel pipettes, filtered pipette tips, clear sealing foil for plates, gloves, permanent marker.

1. Digest 5 µL of Cytb-qPCR amplified products and four positive controls with FspBI or AluI (according to instructions of the manufacturer) to discriminate between *P.falciparum* and *P.malariae* (*P.vivax* and *P.ovale* have an identical band patterns with these enzymes) (Figure 2).
2. All restricted products (samples and controls) should be resolved in 2% agarose (Ultra pure^TM^) with 1X GelRed staining.
3. Run the gels until the bands are separated sufficiently for correct species identification by RFLP.
4. Photograph gel in Bio-Rad Molecular Imager® Gel Doc^TM^ XR+ using the Image Lab^TM^ Software
5. For identification of the different *Plasmodium* species compare the patterns with the respective controls and Fig 2 from original method by Xu et al 2015 [4].
6. Repeat digestion of samples that are unclear or could be *P.vivax* and *P.ovale* with HpyCH4V or Csp6I, or sequencing.

**Figure 2: The cytb-qPCR products and RFLP assays for species determination (Xu et al. 2015).**

**3.3.4 Defining positive samples.**

Flow charts for the molecular screening of samples for *Plasmodium* DNA was used to define positive samples (Figure 3A and 3B). In brief: Pools and single samples were screened with 18-qPCR and/or Cytb-qPCR. Pools/samples were considered positive if positive by either of the two PCR methods. If the cytb-PCR results were unclear, or if the 18s qPCR had a cycle quantification (Cq) value above 40, then the cytb PCR was repeated in triplicate. The samples was deemed positive if any of the three repeats were positive.

**4. References**

1. Group UGH: **Single Low-Dose Primaquine to Interrupt P. falciparum Transmission in Africa: A Roadmap Update.** 2014.

2. Hsiang MS, Lin M, Dokomajilar C, Kemere J, Pilcher CD, Dorsey G, Greenhouse B: **PCR-based pooling of dried blood spots for detection of malaria parasites: optimization and application to a cohort of Ugandan children.** *J Clin Microbiol* 2010, **48:**3539-3543.

3. Kamau E, Tolbert LS, Kortepeter L, Pratt M, Nyakoe N, Muringo L, Ogutu B, Waitumbi JN, Ockenhouse CF: **Development of a highly sensitive genus-specific quantitative reverse transcriptase real-time PCR assay for detection and quantitation of plasmodium by amplifying RNA and DNA of the 18S rRNA genes.** *J Clin Microbiol* 2011, **49:**2946-2953.

4. Xu W, Morris U, Aydin-Schmidt B, Msellem MI, Shakely D, Petzold M, Bjorkman A, Martensson A: **SYBR Green real-time PCR-RFLP assay targeting the plasmodium cytochrome B gene--a highly sensitive molecular tool for malaria parasite detection and species determination.** *PLoS One* 2015, **10:**e0120210.

**
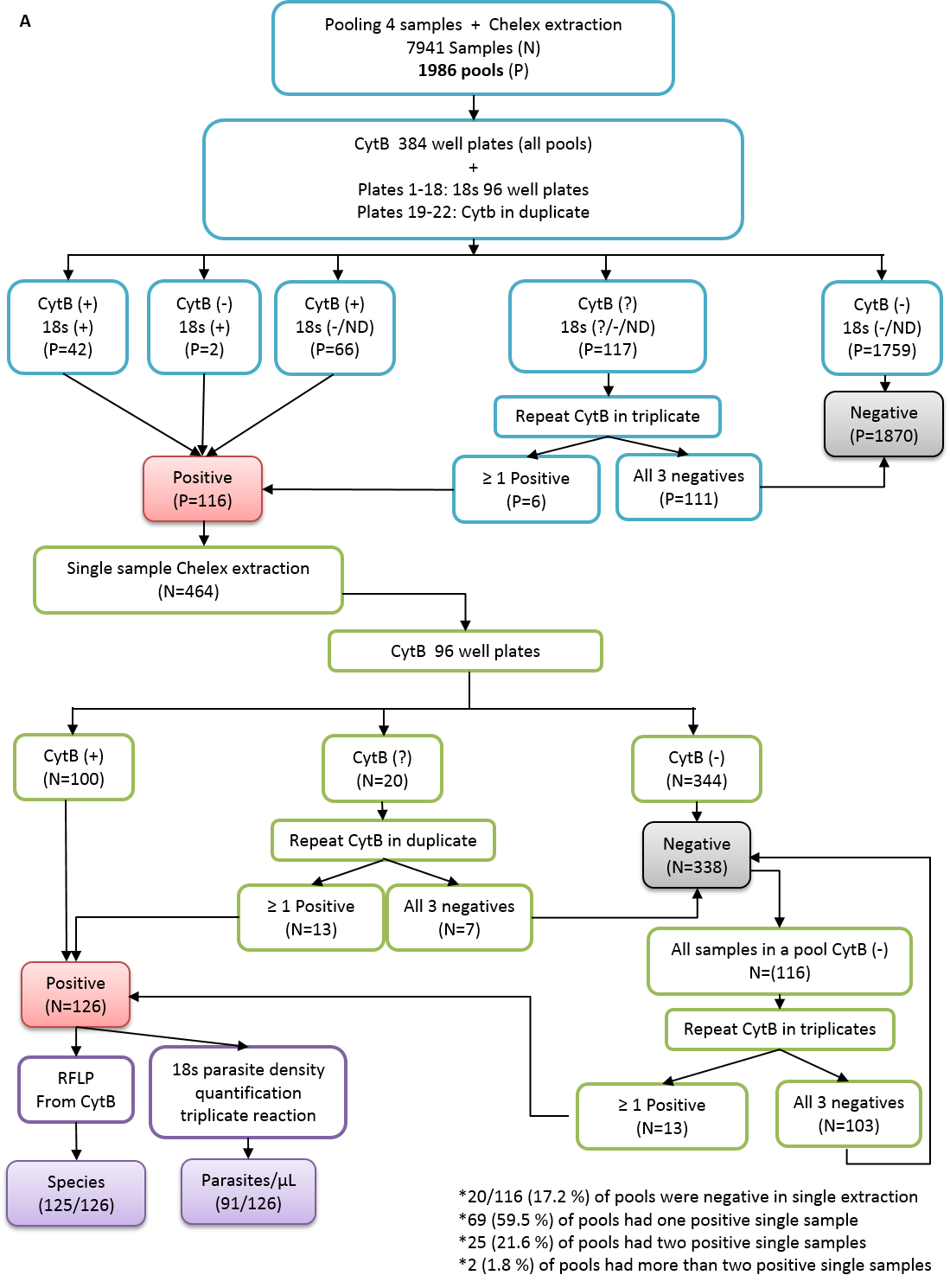
**

**Figure 3A: Flow chart of molecular screening for *Plasmodium* infection in samples collected at the baseline.**

**
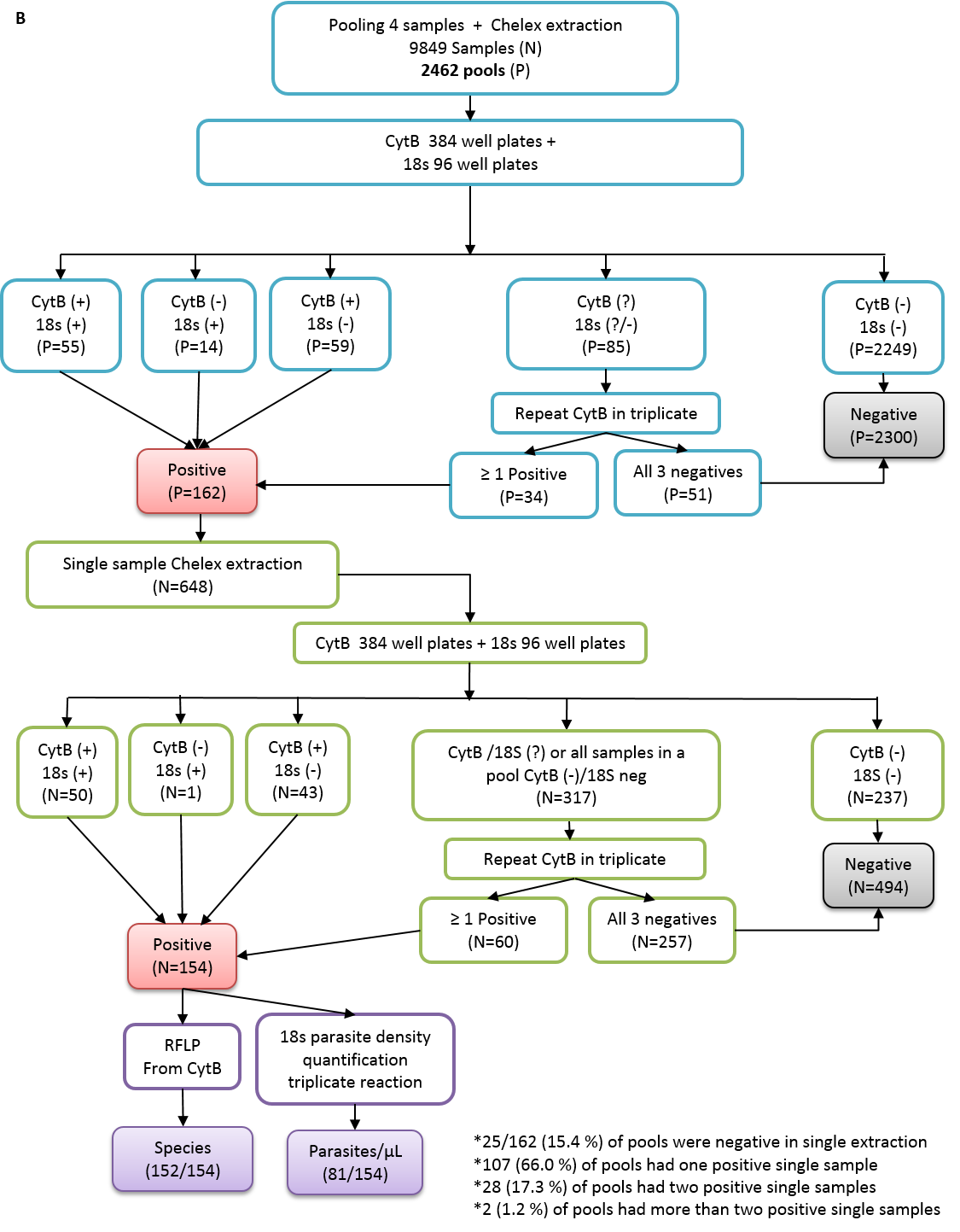
**

**Figure 3B: Flow chart of molecular screening for *Plasmodium* infection in samples collected during the follow-up survey.**
