# Supplementary material for: A cluster randomised controlled trial of two rounds of mass drug administration in Zanzibar, a malaria pre-elimination setting—high coverage and safety, but no significant impact on transmission
Source: BMC Med. 2018 Dec 10;16:215. doi: 10.1186/s12916-018-1202-8 (PMC6287359; doi:10.1186/s12916-018-1202-8)
Supplement: Supplementary file 2 — Figure S1. Capillary whole blood piperaquine concentrations at day 7 post-dose, stratified by self-reported adherence status. Figure S2. Number of clinical malaria cases as reported through the malaria case notification system from January 2015 to Sept 2017 in Unguja. Figure S3. Weekly number of clinical malaria cases as reported in the Malaria Early Epidemic Detections System in Unguja 2016. (PDF 414 kb) [file 12916_2018_1202_MOESM2_ESM.pdf]

Supplementary figures

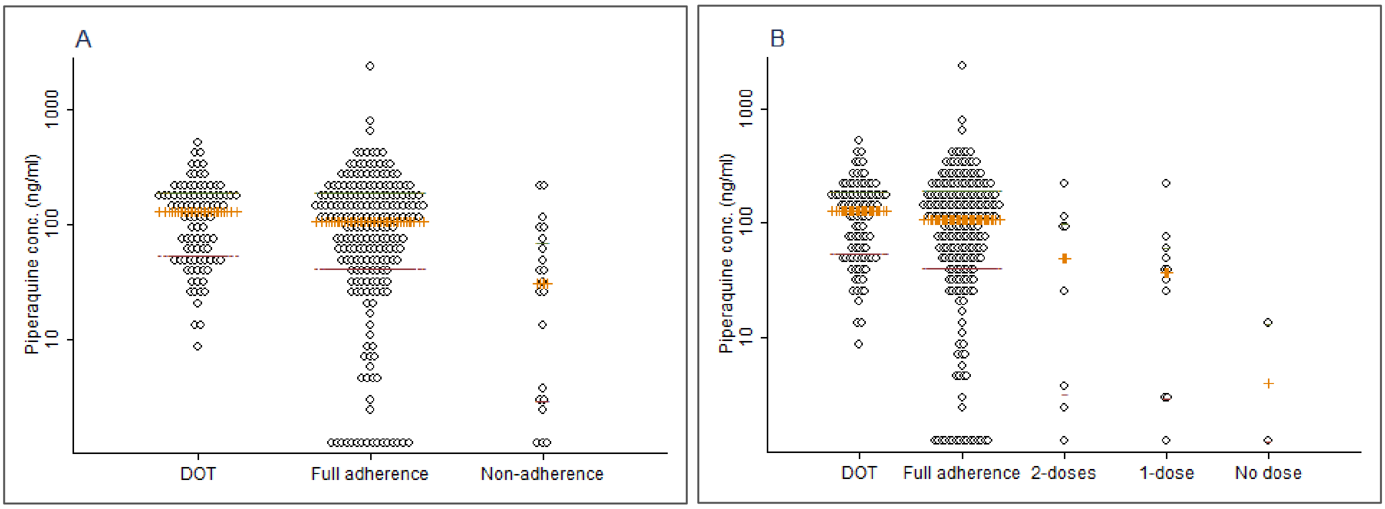

Figure S1: Capillary whole blood piperaquine concentrations at day 7 post-dose, stratified by self-reported adherence status. A) DOT = Observed treatment intake control group. Full adherence = self-reported full adherence to the three day treatment regimen. Non-adherence = self-reported non-adherence to the three day treatment regimen. **B)** 2-dose, 1-dose, and No-doses = number of doses completed in those reporting non-adherence. Dashed yellow lines mark median values. Samples with measurements below the limit of quantification (<LLOQ) were excluded from the observed intake control group (N=10), as well as three samples where vomiting within 30 minutes was reported, and one samples where non-adherence was reported. Measurements below the limit of quantification (<LLOQ) in the non-observed treatment group were imputed as half of the LLOQ (i.e. 1.2 ng/ml).

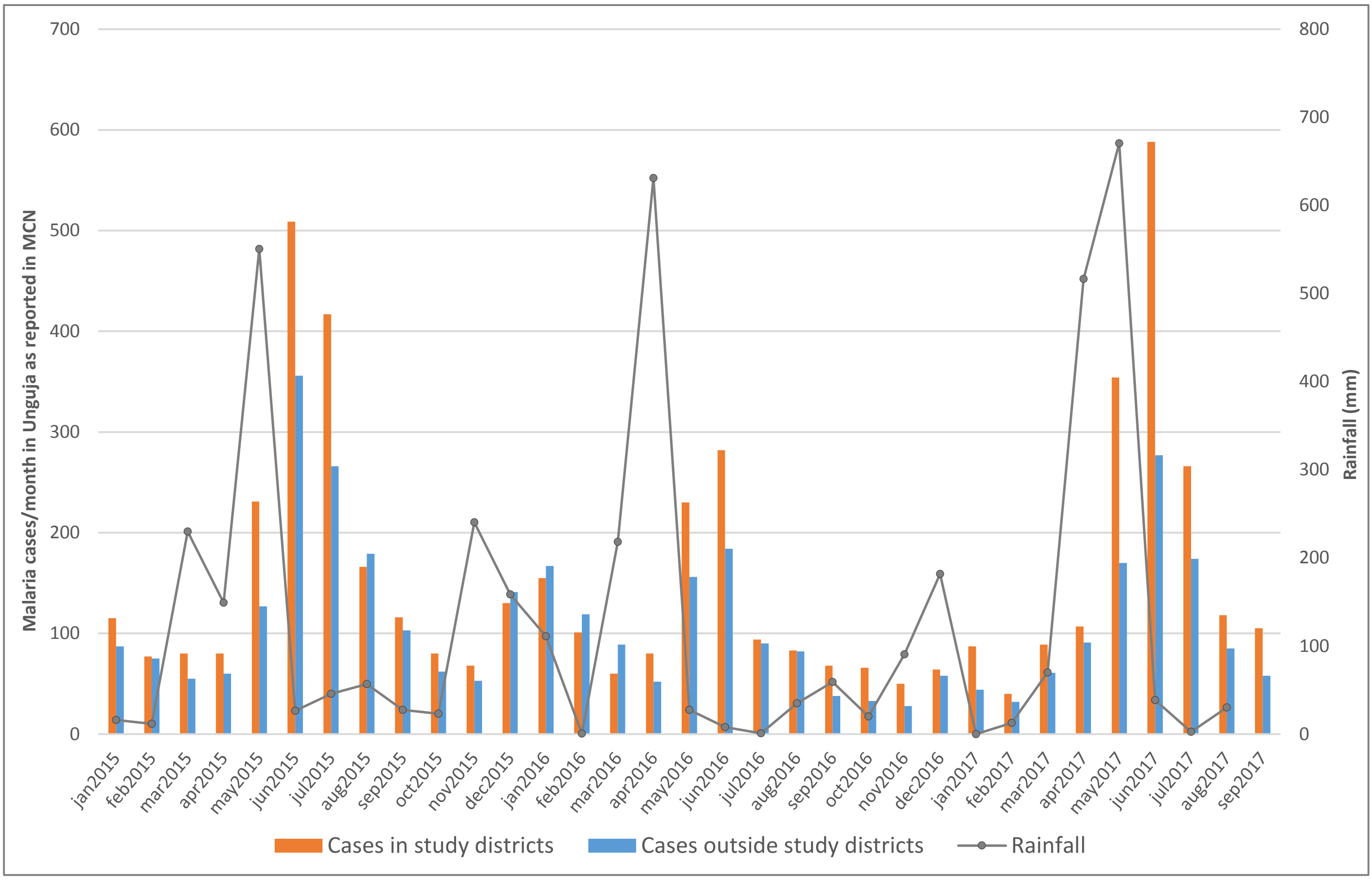

Figure S2: Number of clinical malaria cases as reported through the malaria case notification system from January 2015 to Sept 2017 in Unguja. Bars represent malaria cases reported inside (orange) and outside (blue) the study districts. The line chart represents the monthly rainfall across Unguja Island according to the Tanzanian Meterological Agency Zanzibar Office. There are in total six districts in Unguja. The three study districts were selected for having a larger number of hotspot shehias per district. This is reflected in the larger number of cases reported in the study districts during peak transmission. Since IRS is targeted annually to hotspot shehias, the coverage of IRS may have been greater in the study districts than in the non-study districts. Despite this, the trends in incidence in the study and non-study districts were similar.

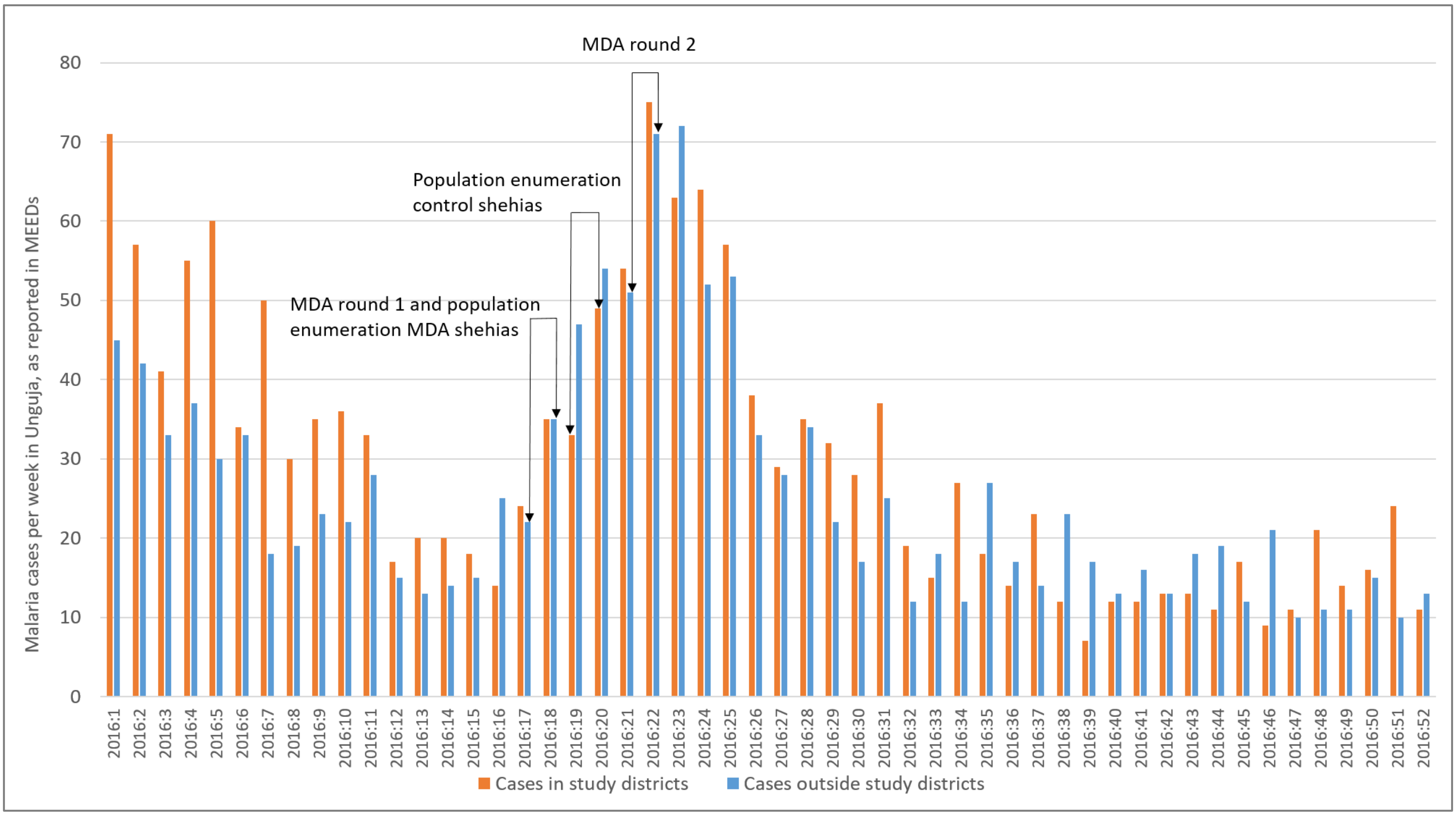

Figure S3: Weekly number of clinical malaria cases as reported in the Malaria Early Epidemic Detections System in Unguja 2016. Bars represent malaria cases reported each week in Unguja 2016 inside (orange) and outside (blue) the study districts. Arrows mark out when population enumeration, and the first- and second round of MDA were conducted, highlighting that the second round of MDA was conducted at peak transmission. In addition, population enumeration and finger prick blood sampling were conducted in the MDA shehias first, so that the second round of MDA would not coincide with the onset of Ramadhan. The figure shows that there had been an increase in transmission when population enumeration and blood sampling were conducted in the control shehias.
